# Distant Site Effects of Ingested Prebiotics

**DOI:** 10.3390/nu8090523

**Published:** 2016-08-26

**Authors:** Stephanie Collins, Gregor Reid

**Affiliations:** 1 Department of Microbiology and Immunology, The University of Western Ontario, London, ON N6A 5C1, Canada; scolli69@uwo.ca; 2 Centre for Human Microbiome and Probiotics, Lawson Health Research Institute, 268 Grosvenor St., London, ON N6A 4V2, Canada

**Keywords:** prebiotics, microbiome, bone, immune, cardiovascular, brain

## Abstract

The gut microbiome is being more widely recognized for its association with positive health outcomes, including those distant to the gastrointestinal system. This has given the ability to maintain and restore microbial homeostasis a new significance. Prebiotic compounds are appealing for this purpose as they are generally food-grade substances only degraded by microbes, such as bifidobacteria and lactobacilli, from which beneficial short-chain fatty acids are produced. Saccharides such as inulin and other fructo-oligosaccharides, galactooligosaccharides, and polydextrose have been widely used to improve gastrointestinal outcomes, but they appear to also influence distant sites. This review examined the effects of prebiotics on bone strength, neural and cognitive processes, immune functioning, skin, and serum lipid profile. The mode of action is in part affected by intestinal permeability and by fermentation products reaching target cells. As the types of prebiotics available diversify, so too will our understanding of the range of microbes able to degrade them, and the extent to which body sites can be impacted by their consumption.

## 1. Introduction

The human colon harbours 10^11^–10^12^ live microorganisms per gram that, along with those in the small intestine, comprise the gut microbiota. In healthy individuals, this vast community acts symbiotically with the host to improve intestinal integrity, metabolism, and compete against pathogenic organisms. Lactic acid-producing *Bifidobacterium* and *Lactobacillus* genera have been regarded for their beneficial effects on the host, notably by their expression of immunomodulatory and pathogen-antagonistic molecules [1]. The targeted metabolism of select compounds by these commensal organisms to provide health benefits has been coined the “prebiotic effect” [2]. Prebiotics are defined as dietary fibers that are selectively fermented by beneficial microbes of the intestine. Unlike probiotics that require administration of exogenous microbes, prebiotics take advantage of the commensals already present in the host to degrade their otherwise indigestible bonds and support microbial survival [2,3]. Of note, it usually requires 5 grams or more to produce sufficient fermentation to benefit the host.

A wide variety of compounds have been studied for their prebiotic attributes. Various chain length oligosaccharides are the most common, including fructo-oligosaccharides (FOS) and galacto-oligosaccharides (GOS)/trans-galactooligosaccharides (TOS). FOS are found in several naturally occurring foods including artichokes, asparagus, garlic, and wheat. Their structure consists of a terminal glucose residue connected to a chain of β(2,1)-linked fructose residues. Inulin refers to highly polymerized FOS, containing at least 10 sugar monomers, and anything shorter is termed oligofructose. Also terminated by a glucose monomer, GOS/TOS comprise repeating units of galactose linked by β(1,4) or β(1,6) bonds, usually 2–8 residues in length. Other less common oligosaccharides include polydextrose/β-glucans, which are linear or branched polymers of glucose, chains of xylose monomers denoted as xylo-oligosaccharides (XOS), and pectin-derived acidic oligosaccharides (pAOS), which are undefined oligosaccharide structures formed by partial enzymatic digestion of plant pectins [4]. Tri-, di- and even monosaccharides may be used as prebiotics, as long as they contain host-indigestible bonds. Some examples are raffinose, lactulose, and monomers l-rhamnose and arabinose. Lastly, other atypical compounds such as sugar alcohols (e.g., lactitol) and the cyclic disaccharide di-fructose anhydride III have been used as prebiotics.

Depending on the structure and combination of prebiotic compounds administered to the individual, effects on the microbiome will vary. In general, the selective fermentation of prebiotics by bifidobacteria and lactobacilli will increase numbers of these commensals while displacing other neutral or pathogenic organisms, overall supporting a symbiotic gut microbiota composition [5]. Additionally, this process alters the metabolism and activity of gut microbes, often lowering colonic pH due to acidic fermentation products such as lactate and short chain fatty acids (SCFAs) butyrate, acetate, and propionate. The fermentation products preferentially produced by these microbes depend on the prebiotic’s structure and the bacterial communities present [6,7], since some organisms utilize by-products from the degradation of more complex carbohydrates [8]. In turn, this determines what effects the prebiotic will have on host health in the gut and at distant sites.

Butyrate is known to exhibit beneficial properties in the gut since it supports the growth of intestinal epithelium, but its benefits are not limited there [9]. Butyrate and other SCFAs are considered the enactors for prebiotic effects elsewhere in the body due to their ability to pass through enterocytes into circulation [10]. This is crucial for highly polymerized or branched oligosaccharides that may only pass through epithelium in small amounts when highly concentrated in the intestinal lumen [11]. Ultimately either the prebiotic or its metabolites are required to enact changes apart from the gastrointestinal system, but there remains much more to be known about how these molecules specifically act upon the immune, circulatory, and neural systems. For this reason, research on prebiotics’ influence on human health is burgeoning with exciting new findings.

Therefore, the purpose of this review is to examine the current evidence of how prebiotics impact distant sites, what is known about their mechanisms of action, and what potential exists to exploit these applications.

## 2. Bone Density and Strength 

Osteoporosis is a condition that reduces bone density and strength, thereby increasing risk of fracture. It is especially prevalent in postmenopausal women and is magnified with age, as osteoporosis affects approximately 10% of women aged 60 up to almost 70% of women aged 90 [12]. To counter this, women are encouraged to increase their intake of minerals such as calcium and magnesium to counter bone loss. However, the finding that SCFAs modulate nutrient absorption and intestinal permeability [8] has opened up the possibility of using prebiotics to enhance calcium and magnesium absorption from the intestinal lumen, ultimately increasing bone regeneration and preventing osteoporosis.

Several rat models have demonstrated that GOS intake improves calcium absorption, retention, bone density, and strength [13,14]. The most notable benefits were reported in ovariectomized (OVX) rats, a model for the hormonal changes at menopause, which are a key risk factor for developing osteoporosis. Feeding these rats 5.0–5.5 g/100 g inulin and oligofructose daily for several weeks restores calcium and magnesium absorption capabilities and positively affects indicators for bone health such as strength and density [15,16]. More acutely, polydextrose can also enhance calcium and magnesium absorption [15]. Both long and short term bone benefits have been observed for OVX rats fed 5% per weight GOS, including improved calcium uptake into circulation and bone, and femur and tibia weights [17]. Another non-digestible compound not typically considered for prebiotic usage, di-fructose anhydride III, at a mere 3% supplement to mouse diet, improved bone mineralization, calcium and magnesium uptake and bone strength in OVX rats after four weeks [18,19].

In human studies, failure to account for variation in diet and utilize a reasonably homogenous cohort, has meant that effects are much less pronounced. Nevertheless, such studies have shown mostly positive changes in mineral absorption [20,21,22]. A summary of human prebiotic trials for bone health is shown in Table 1. Postmenopausal women have been the focus of many human trials because of their particular risk in experiencing bone loss. Oligofructose-inulin blend has been shown to improve calcium and magnesium absorption, distinctly in individuals whose bone density was low prior to treatment [23]. However calcium uptake was not benefitted by oligofructose consumption in a study of early menopause, possibly because of the small sample size of 12 and large variation in mineral absorption at this stage of menopause [20,24]. Another study observed a 42% increase in calcium absorption following 3 months of chicory fructan administration [25], while TOS and lactulose have enhanced calcium uptake by 16% [26,27].

Adolescents and young adults also require successful mineral uptake and bone formation because of their rapidly growing bodies. An early study of teenage boys consuming 15 daily grams of inulin, FOS, or GOS observed no differences in calcium absorption after 3 weeks [28]. Scholz-Ahrens et al. [22] suggested that the lack of significant improvements in circulating calcium could be attributed to either insufficient dosage or time allotted before urine collection. Since prebiotics are typically not digested by the host or bacteria in the small intestine, but are instead fermented in the colon, it takes greater than the typically employed 24 h for prebiotics to reach and interact with colonic microbes. As a proof of principle, consumption of oligofructose and inulin-type fructans by teenagers, allowing at least 36 h of induction before final samples were taken, significantly improved calcium absorption [29,30,31].

In terms of the applicability of this enhanced uptake of bone minerals to actual improved bone density and health, the results are contentious. In two of the studies mentioned above on postmenopausal women, markers of bone remodelling and formation are unchanged or reduced with FOS, despite improving mineral uptake [24,25]. In contrast, a recent trial concluded that long-term FOS consumption has no effect on bone density but improves turnover, especially in women with osteopenia [32]. Similarly to another trial, only individuals with improved mineral uptake had improved bone metabolism in this cohort [23], suggesting that at-risk individuals are deficient in their ability to absorb calcium. It would be worth assessing the gut microbiota and barrier function of responders and non-responders to see if this explains the differences [33]. Interestingly, prebiotic interventions for bone density might be more effective in children or young adults than postmenopausal women, given that 8 g daily inulin for one year significantly increased bone mass and density throughout the teenage skeleton [29]. This makes a case for recommending prebiotics for individuals at risk for bone loss, but more human studies are needed to confidently observe benefits and determine the duration and extent of these changes.

Numerous mechanisms have been described to explain prebiotic effects on bone health. Colonic fermentation of prebiotics by organisms such as bifidobacteria [14] result in the release of SCFAs and subsequent drop in pH. This SCFA-induced acidic environment, as well as GOS, lactulose, and inulin ingestion in general, are associated with enterocyte generation and improved colonic surface area, thus a greater capacity to absorb minerals [22,34,35]. Negatively-charged SCFAs also conjugate with Ca^2+^ ions to enhance passive diffusion through the lipid membrane of enterocytes, and their capacity to donate a proton can be used for exchange with luminal calcium through H^+^/Ca^2+^ antiporters [36,37]. These effects may become limited with age due to the decline of bifidobacteria numbers [38], perhaps correlating with reduced uptake of calcium and magnesium. Yet, it seems hard to imagine that a single bacterial genus plays such a pivotal role in skeletal strength, and the reason for its decline is unknown. It is more likely that a number of functionally redundant gastrointestinal bacteria must become reduced to impact bone density and health. Since there is little to suggest that intake of microbiome-stimulating fermented foods or fibres lowers in elder years, at least per a Korean study [39], dietary and drug intake patterns should be queried to try and understand how the microbiome changes with age to affect bone health. For example, proton pump inhibitors disproportionately used by older adults induce dysbiosis notably by a loss of bifidobacteria [40], but are only weakly associated with fractures and reduced bone density [41,42]. Other medications used predominantly by the older population such as diuretics [43] and glucocorticoids [44], used to treat water retention and inflammation respectively, are independent risk factors for osteoporosis and bone density loss [45].

## 3. Prebiotics and the Brain

The central nervous system and the gut, including its indigenous microbes, are bi-directionally linked by the “gut-brain axis”, a network of distinct connections between these two major systems [46]. Several reviews have extensively covered the topic of microbiome effects on the brain [46,47,48], and concluded that three main pathways are involved—neural, endocrine, and immune. The major afferent neural pathway by which microbial products influence the brain is the vagus nerve, which directly innervates the gut [49]. Bravo and others [50] showed that the presence of certain gut microbes influences GABA receptor expression in the brain, and this link is lost by severing the vagus nerve. Prebiotics such as FOS and GOS, including the patented GOS produced by Bimuno (B-GOS), likely act through this connection to modulate neural growth factors like brain-derived neurotrophic factor, neurotransmitters such as d-serine, and synaptic proteins including synaptophysin and NMDA receptor subunits [51,52].

The primary neuroendocrine pathway affected by gut microbes is the hypothalamic-pituitary-adrenal (HPA) axis. This was determined by Sudo et al., who observed that normal microbiome development in mice is required for the induction of an appropriate stress response involving corticosterone and adrenocorticotropic hormone (ACTH) [53]. It is not surprising, then, that GOS and other prebiotics are able to modulate hormones such as plasma peptide YY [51]. Immune related effects also play an important role, but will be discussed in a later section. Together, studies on these systems have emphasized the necessity of gut microbes for the regulation of brain health. This has provided an avenue for prebiotics to manipulate neural processes, including cognitive ability, mood, and prevent certain psychological disorders through changes in microbiome activity and composition [48].

### 3.1. Memory, Attention, and Learning

Throughout our lifetime, memory and learning are crucial for mental health. This is especially apparent when we experience head trauma, Alzheimer’s disease, or dementia, where these abilities are stunted. Many recent studies have linked the consumption of fermentable compounds to synaptic plasticity and memory, both in animal and human trials [54]. In rats, a polydextrose/GOS mixture and oligofructose-enriched inulin both improve memory by novel object recognition tasks or light extinction test [55,56]. A study by Sakai et al. [57] also demonstrated improved learning in rats administered sialyllactose and galactosylated *N*-acetylneuraminic acid. However, this may be specific to sialyllactose metabolism since its component sialic acid is known to enhance memory alone [58]. The story in humans is also consistently positive, as summarized in Table 2. Although administration of oligofructose-enriched inulin has no long-term benefits, possibly due to the numerous confounding factors in memory retention compounded over time, acute memory is significantly improved after a single 5 g dosage of oligofructose-enriched inulin [59,60]. A series of experiments on middle-aged adults by Best et al. [61,62] consistently demonstrated memory improvements due to various prebiotics. In two studies, Ambrotose complex, a mixture of arabinogalactan, aloe vera extract, and gum components, improved memory performance in the Rey Auditory Verbal Learning Test, even when controlling for blood glucose. Furthermore, regular dietary intake of foods containing indigestible carbohydrates such as rhamnose, xylose, and mannose may be sufficient to see improved memory aptitude, even if these effects are only self-perceived [63].

Due to the exceedingly promising results of these studies, it is important to determine how prebiotics could attenuate dementia and other conditions affecting memory. Using a mouse model of vascular dementia, Han et al. [64] demonstrated that arabinoxylan and arabinose improve general cognition, as measured by quicker completion of maze, and can limit dementia-related accumulation of glial fibrillary acidic protein. Other populations, such as preterm babies, also tend to be stunted in their memory and learning abilities due to underdevelopment. However, in a trial of 77 preterm infants, scGOS/lcFOS/pAOS had no effect on neural generation after two years [65]. Thus, prebiotics may be more effective in maintaining recall and learning rather than development.

### 3.2. Mood and Anxiety

A pioneering study equating the gut microbiome to mood and stress was performed by Sudo et al. [53]. They found that germ-free mice exposed to restraint stress had significantly elevated levels of circulating stress hormones ACTH and corticosterone, which were later shown to translate directly to anxiety-like behaviour [66,67]. Interestingly, normal ACTH and corticosterone levels could be restored by administering *Bifidobacterium infantis* to these otherwise sterile mice [53]. Given their bifidogenic properties, there is therefore sound basis for studying the stress-relieving potential of prebiotics. 

Several animal models used in anxiety-related prebiotic trials have further clarified this connection. Administration of polydextrose and GOS to mice and male piglets was shown to reduce anxiety-related behaviour, and in rats, improve positive social interactions [56,68]. These effects may be partially attributable to structural changes in the development of the brain. For example, prebiotic-supplemented piglets have less gray matter compared to those fed a control diet, indicating an improvement in neural pruning [68]. This was also linked to beneficial intestinal enzymatic activity and modified gut microbiota [69]. There is still a lack of understanding of how these microbial changes and memory effects of prebiotics are connected, though some prebiotics have a well-defined action on stress responses because of known structure-specific effects. Digestion of 3′ and 6′ sialyllactose releases lactose and sialic acid, where the latter acts directly on the brain to improve memory and learning [58]. In further support of a prebiotic-independent effect of these compounds, Tarr and others [70] showed that mice fed sialyllactose had less anxiety in response to stressors without changes to the microbiome. However, without examining the metabolome, fermentation-induced changes to microbial activity cannot be ruled out.

The current lack of human studies in this realm is partially a result of the complications and convoluting factors in studying behaviour and anxiety in humans, which have historically been avoided by directly measuring stress hormone levels. One group reported that daily consumption of 5.5 g of B-GOS by healthy individuals limited rapid elevations in salivary cortisol and improved attention upon exposure to a negative stimulus (Table 2) [71]. Further development in this field will require more human subjects, but considering the strong foundation by pioneering studies in animals, these appear to be promising. However, studies must be approached with particular caution due to newfound ethical concerns with manipulations of the microbiota. The observation that exploratory behaviour may be transferred with fecal transplants from normal to germ-free mice [72] emphasizes the importance of considering the psychological effects of altering gut microbes. While the intent of modulating the gut microbiota is to benefit people with brain and mental health ailments, one should be cautious in transferring personality traits between humans. 

### 3.3. Autism Spectrum Disorders

One of the most apparent links between gastrointestinal and psychological health is in individuals with autism spectrum disorders (ASDs). Rates of gastrointestinal disorders in persons with ASDs have been reported to affect up to 70% compared to 9% of otherwise healthy individuals [73], with seriousness of gastrointestinal symptoms increasing with autism severity [74]. Gut microbes are an important factor contributing to digestive problems in individuals with ASDs, since the condition has been linked to a fecal microbiome with unusually high levels of *Clostridium* and depleted *Bifidobacterium* [74,75]. Several prebiotics including the wheat fiber Nutriose^®^, among their benefits to colonic acidity, reduce *Clostridium perfringens* and enhance bifidobacteria [76], making prebiotics a promising therapeutic for ASD dysbiosis. The gut metabolome of children with autism also differs from healthy children, with characteristically reduced SCFAs [74]. Although probiotics have not successfully counteracted this depletion [74], the ability of prebiotics to stimulate SCFAs makes these products worthy of pursuit. However, the selection of prebiotics by their SCFA products becomes complicated by the recent implication of propionate and butyrate in autism pathogenesis. These SCFAs were found to increase the expression of tyrosine hydroxylase in a rat adrenal medulla cell line. This enzyme is responsible for producing catecholamines, a class of neurotransmitters whose levels are higher in individuals with ASDs [77]. It is not currently known if this study is translational to human subjects, but researchers selecting prebiotics for use on ASDs should consider compounds that avoid the production of these SCFAs. Ultimately, due to the lack of direct studies testing prebiotics in humans and the elusiveness of the condition, much more evidence is needed before conclusions can be made about prebiotic effects on ASDs. Furthermore, the finding that *Bacteroides fragilis* treatment of mice had no effect on microbiota richness, evenness, or relative class-level abundance, despite improving ASD behaviour [78], emphasizes the need to carefully analyse microbiome data [79] before selecting probiotic, prebiotic or synbiotic interventions. Understanding which prebiotics could stimulate specific SCFAs, excluding propionate and butyrate, might better target ASD therapy.

### 3.4. Hepatic Encephalopathy

Hepatic encephalopathy is a devastating mental condition brought about by failure of proper liver functioning. This causes rapid deterioration of a range of neural processes from movement and speech to cognition and personality, sometimes leading to coma and death if left untreated. Though its etiology is not entirely clear, serum ammonia levels are directly predictive of encephalopathy severity, and are considered the main enactor of neural degradation in the condition [80]. An incredible success in the treatment of hepatic encephalopathy occurred in 1966 with the introduction of prebiotic lactulose to manage gut ammonia [81]. Since its employment, lactulose has been widely regarded for its broad benefits to cognition and quality of life in encephalopathy patients [82]. Two recent meta-analyses of randomized human trials concluded that lactulose can effectively prevent and treat hepatic encephalopathy, and also improve underlying liver dysfunction [83,84].

Early studies recognized several probable mechanisms of lactulose’s benefits on elevated blood ammonia. Fermentation of lactulose into fatty acids such as lactate reduces fecal pH by releasing protons into the colonic lumen. It was proposed that these protons convert ammonia into ammonium to create a concentration gradient that increases ammonia reuptake from blood into the gastrointestinal tract [85]. Concurrently, lactulose provides fermentable energy to colonic bacteria, diverting them from producing ammonia by amino acid metabolism, and can even directly inhibit glutaminases which degrade glutamine into ammonia [83]. Lastly, lactulose can flush out metabolites such as ammonia by shortening colonic transit time. Many of these properties are shared by a number of prebiotics—colonic acidification, fermentation, and stool softening—and have provided the basis for testing other indigestible saccharides for this purpose. In fact, a number of other compounds can also benefit hepatic encephalopathy, particularly lactitol [86]. Lactitol is equally as effective as lactulose in treating encephalopathy, and actually has fewer negative side effects such as diarrhea [87,88]. The success of using these prebiotics for treating a liver and brain related disorder further provides evidence for prebiotic compounds impacting distant sites.

## 4. Immune Function

It has been well documented that prebiotics can modulate immune functioning locally in Peyer’s patches, but an increasing body of evidence suggests that these compounds also exert immunological benefits throughout the body. Although isolated immune systems are generally unaffected by prebiotics such as fructans [89,90], clear benefits are observable in response to immune challenges. Since we are constantly exposed to a range of foreign stimuli, prebiotics could therefore be effective to downplay their effects. We will review here the immunomodulatory benefits of prebiotics in the context of challenges from pathogens, atopic dermatitis, and chronic inflammation, as well as their ability to improve mounted responses against vaccinations. Key human interventions studying prebiotic benefits on immune function will be summarized in Table 3.

The immunostimulatory properties of prebiotics can, in some cases, improve mounted memory development against vaccinations and pathogens. Oligofructose administered to infants several weeks leading up to measles vaccination elevates their measles-specific IgG response [91]. Even later in life, β-fructan prebiotics promote adaptive responses against influenza vaccination. In a study by Lomax et al. [92], four weeks of 8 g daily oligofructose/inulin mix prior to and following immunization was sufficient to increase circulating antibodies for influenza in a cohort of middle-aged adults. Unfortunately, this mounted immune response was limited to the H_3_N_2_-like strain, despite the vaccine also containing H_1_N_1_- and B-like virus. A similarly selective response was seen in a cohort of elderly persons consuming a dietary supplement containing oligofructose/inulin and triacylglycerol, with antibodies mounted for H_1_N_1_- over H_3_N_2_- and B-like viruses [93]. It is currently unknown what properties of the prebiotics impart this selectivity, though if this were determined, prebiotic formulations could be manipulated to stimulate unique immune responses against immunized viruses. Prebiotics may also have general immune benefits when used alongside immunization. A unique oligosaccharide mixture (9:1 short-chain GOS to long-chain FOS + pAOS) fed to mice prior to vaccination improved their type IV hypersensitivity response, a form of cell-mediated immunity [94]. In turn, this immune response evoked enhanced skin induration and CD4+ T cell counts against toxins other than the vaccinated pathogen [95]. If this is observable in humans, an improvement in circulating antibodies and cell-mediated immunity by a food product would be beneficial especially to individuals at high risk for infection such as children and the elderly.

Prebiotics and probiotics have been extremely successful at suppressing indigestion and diarrhea due to pathogens. These conditions in infants are common and can be quite serious, particularly in the developing world [96]. For this reason, researchers have measured the effects of oligofructose on diarrhea-associated fever and required medical attention in infants [97,98]. They found that children with a similar occurrence of diarrhea experienced fewer episodes of fever, trips to the doctor, and antibiotic use while consuming 1 g/day oligofructose-supplemented cereal. Even though their ability to combat loose stool was unchanged, oligofructose may improve young children’s ability to fight off underlying infection to reduce associated morbidities.

On the other end of the immune spectrum, excessive response to a foreign stimulus can lead to allergic diseases such as asthma, atopic dermatitis, and eczema. A meta-analysis of human clinical trials for allergic diseases in children concluded that prebiotics are protective against the onset of eczema, with a potential trend for combatting allergic diseases [99]. For example, infants fed formula supplemented with a GOS/FOS/pAOS mixture for 6–10 months had a 44% lower prevalence of atopic dermatitis and a low risk for developing the condition [100], as did another formula containing a GOS/FOS mixture [101]. Contrary to this, a more recent study used partially hydrolyzed formula supplemented with the same GOS/FOS prebiotic and found no effect on eczema prevalence [102]. However, partially hydrolyzed formula components such as whey protein can minimize allergic skin reactions alone, and this may have masked the effects of prebiotic supplementation [103]. Even naturally occurring human milk oligosaccharides found in women expressing the sugar secretory protein FUT2 are associated with fewer cases of allergic disease and eczema in their breast-fed children [104]. This is partially due to fructan’s inhibitory effect on IgE expression, such as what was observed in a synbiotic trial by Kukkonen and group [105]. Others postulate that galectin-9 may be upregulated by milk oligosaccharides to induce T cell differentiation selectively into Th1- and regulatory T cells, which mitigate excessive inflammation [106]. Support for this hypothesis also comes from the fact that depletion of CD25+ regulatory T cells reverses the beneficial effects of scGOS/lcFOS/pAOS treatment on atopic dermatitis in mice [107]. Both of these direct and indirect mechanisms are likely involved in the action of milk oligosaccharides on suppressing excessive immune responses.

Though there are few human trials for the use of prebiotics to prevent allergic diseases other than atopic dermatitis, support can be found from animal studies. In an ovalbumin (OVA)-induced mouse model for allergic asthma, scGOS/lcFOS reduces symptoms including bronchial inflammation, skin lesions and lung resistance, probably through a reduction in IgE and increased OVA-specific IgG and regulatory T cells in serum [108,109]. Unfortunately a small proportion (0%–3.5%) of the population is allergic to GOS, though this is dependent on the particular formulation [110]. More research will be needed to take these promising results into clinical testing and select a prebiotic with the least allergic properties, particularly in children at risk for developing allergic asthma.

While there is a clear link between inflammation and allergic diseases, long-term inflammation more subtly contributes to a number of chronic problems such as cancer, cardiovascular, and lung diseases. There is, therefore, a growing interest in modulating immune responses to decrease long-term inflammation. Elderly people, who are generally most affected by the comorbidities of chronic inflammation, also commonly experience gut dysbiosis with a decreased proportion of bifidobacteria [111]. A number of studies have shown that prebiotics can benefit the elderly by improving their gut microbiota and immune function, ultimately reducing inflammation. An 8 g daily oligofructose/inulin mixture given for three weeks to the elderly reduced expression of pro-inflammatory IL-6 by peripheral blood mononuclear cells (PBMCs) and improved T cell counts [112]. Others have observed that B-GOS (5.5 g daily for 10 weeks) lowers circulating levels of inflammatory cytokines such as IL-6, IL-1β, and TNF-α, and raises immunomodulatory IL-10 [113,114]. Furthermore, B-GOS stimulates natural killer T cells, potentially improving antiviral activity [113].

Distant effects of prebiotics on the immune system are likely mediated by fermentation products of gut bacteria that pass through enterocytes to tissue and circulating immune cells, or in rare cases, direct binding of prebiotics to immune cells. As an example, SCFAs, especially butyrate, derived from microbial degradation of inulin, hi-maize and β-glucan can reduce production of IFN-γ, IL-12, TNF-α, and TGF-β1, and elevate IL-4 and IL-10 release from lipopolysaccharide-stimulated PBMCs [115]. It has been postulated that IL-10 acts as an overarching mediator of anti-inflammatory effects on a range of cytokines. This is not the complete story for all prebiotics, since soluble dextrins can reduce proinflammatory cytokines even in the absence of IL-10 [116], however in most cases, IL-10 is elevated following prebiotic administration. Alternatively, some indigestible carbohydrates may actually permeate through the colonic barrier and act on immune cells by binding sugar-specific receptors. The well-characterized dectin-1, a monocyte and macrophage receptor protein, has particular affinity for β-glucan prebiotics [117,118]. Binding of β-glucan activates this receptor to adhere to pathogenic *Candida albicans* and induce phagocytosis, thereby improving antimicrobial properties in the liver, lung, and thymus, where this receptor is highly expressed [117].

The key to future studies will be fully characterizing the distinct mechanisms employed by different prebiotics to balance immune responses, particularly due to the broad range of modulatory activities they exert. For this reason, prebiotics are an exciting therapy for counteracting dysregulation of the immune system linked to the microbiome.

## 5. Dermatological Health 

New and upcoming applications of prebiotics have emerged for a variety of skin-related conditions. This was first recognized by researchers’ observations that prebiotics can attenuate allergic-related skin diseases such as atopic dermatitis, as discussed in the previous section. However, a few other studies have shown alternative benefits to dermatological health. In particular, UV radiation is damaging to the skin, leading to erythema and sometimes cancer. In hairless mice, a model for human skin, 12 weeks of GOS supplementation improved water retention and prevented erythema [119]. Furthermore, GOS treatment increased dermal expression of cell adhesion and matrix formation markers CD44, TIMP-1, and type 1 collagen, thereby improving the skin’s barrier properties. Even in the absence of UV radiation, hydration and keratinization are critical for healthy skin. In women, GOS alone or with probiotic *Bifidobacterium breve* can prevent water and keratin depletion caused by phenolic compounds [120]. Phenols such as p-cresol are normally produced by gut microbes as a by-product of aromatic amino acid metabolism, which can be absorbed and transported into the skin [121] and are toxic in cases of renal failure [122]. The beneficial effects of GOS on skin character could therefore be attributable to their ability to divert enteric microbes from amino acid metabolism and production of damaging phenols by providing an alternative food source.

## 6. Cardiovascular Health

In 2013, approximately 30% of deaths in the US were a result of cardiovascular disease, including stroke and heart failure [123]. The major contributors to this alarming statistic are poor nutrition and the rise in severe obesity, particularly in the young population [123]. A diet high in fat and low in fiber is increasingly popular in the developed world, and has therefore enticed researchers to evaluate how eating habits, and food-grade compounds such as prebiotics, can be utilized to reduce cardiovascular risk and other obesity-related comorbidities. As described earlier, a component of this is the ability of prebiotics to alleviate chronic inflammation thereby lowering the risk for developing cardiovascular disease. Although direct influence of prebiotic intake on cardiovascular health has not been shown, a number of studies have associated indigestible fiber intake with improved serum lipid profiles, as summarized in Table 4. Targets for this therapy are to reduce blood triacylglycerol (TAG) and low density lipoprotein (LDL) cholesterol, and elevate high density lipoprotein (HDL) cholesterol that become imbalanced in heart disease. Thus prebiotics that improve the equilibrium of these fats may be beneficial as part of an effective nutritional intervention to combat cardiovascular risk.

The vast majority of human lipid profile and prebiotic studies have utilized fructooligosaccharide compositions containing oligofructose and inulin, but the results have been contentious. Letexier et al. [124] observed a reduction in serum TAG and lipogenesis in the liver of healthy adults with a three-week regimen of 10 g/day inulin. Yet a nearly identical study by Forcheron and Beylot [125] saw no significant changes to lipid profile or synthesis in a larger cohort, despite a much longer 6 month intervention. This might be because an inulin-oligofructose mixture, rather than pure inulin, was used, however oligofructose requires the same enzymes for microbial metabolism and should therefore have similar effects on the microbiome. In another study, the potential benefits of inulin on plasma TAG, HDL, and lipoprotein(a) were confounded by the administration of a regulated carbohydrate-rich, low-fat diet. Compared to baseline, inulin-supplemented diet balanced the lipid profile, but following the interventions, no significant differences were observed between inulin and placebo diet except for a reduction in the total cholesterol/HDL ratio [126]. This underlines the importance of controlling for other factors such as diet in prebiotic research. A meta-analysis of human clinical trials between 1995 and 2005 identified the TAG-reducing properties of fructooligosaccharides, with a 7.5% average decrease in serum [127]. The author admitted that this was a minimal reduction compared to current drug therapies, and that only 5 out of 16 studies actually reached significance. Since this meta-analysis, several other studies have corroborated this trend where TAGs are significantly lowered by a small proportion of interventions, enough to cause a mean effect [128]. It could be that large reductions in TAG are observed in some cases and no change in others because of differences in the initial concentration of these fats in the population studied. Presumably larger decreases would be observed in individuals with initially high levels of TAG.

To a lesser extent, a number of other prebiotic compounds have had positive effects on serum lipids. High dosages (25 g/day) of l-rhamnose and lactulose have been shown to minimize both the synthesis and serum concentration of TAG [129]. This is in contrast to results from another study observing that lactulose elevates circulating cholesterol by 10% and apolipoprotein B by 19%, potentially negating its beneficial effects [130]. In overweight subjects, a 12-week treatment of 5.5 g/day B-GOS reduced serum triglycerides and total:HDL cholesterol ratio and modulated circulating insulin, though the effect was far more pronounced in men than women [131]. The benefits of B-GOS on plasma lipids is likely limited to individuals with hypercholesterolemia, since identical dosage to healthy elderly adults saw no effect on total or HDL cholesterol [113]. A more generalizable effect has been observed with consumption of β-glucan prebiotics. A recent meta-analysis of 126 β-glucan studies concluded that total and LDL cholesterol are slightly reduced, an average of 0.60 mmol/L and 0.66 mmol/L respectively, given at least 2 g daily supplementation [132].

An important consideration with these interventions is that, although each prebiotic was bifidogenic, there was a large variability between compounds on which serum lipids were modulated upon treatment and to what extent. Several researchers have proposed that the profile of SCFAs produced by gut fermentation of each prebiotic directs its effect on lipid synthesis and circulating concentrations. For example, absorbed acetate is converted to acetyl-CoA where it acts as a substrate for fatty acid synthesis in hepatocytes [133], explaining why cholesterol and triglycerides in blood are increased by rectally infused acetate [134]. Thus, predominantly acetate-producing substrates such as lactulose [135] and GOS [6] may have a negative effect on lipids when ingested. Fructooligosaccharides tend to produce relatively equal proportions of acetate and butyrate, potentially balancing this effect [6]. Interestingly, propionate prevents acetate-induced lipid synthesis [136], suggesting that propionate producers such as l-rhamnose and fructooligosaccharides could balance the lipogenic effects of acetate [6,135]. Careful selection of prebiotics by their acid metabolites for management of cholesterol and other serum lipids is ultimately of utmost importance to obtain optimal results.

With the success of certain prebiotics in balancing the serum lipid profile and promoting satiety, these fermentable sugars may also benefit other obesity-associated comorbidities. For this reason, prebiotics have been proposed as a treatment for fatty liver disease, despite there being insufficient human trials utilizing only prebiotics to support this claim [137]. An ongoing randomized, placebo-controlled trial will help elucidate mechanistic links and potential benefits of administering an oligofructose-inulin mixture to NAFLD patients [138]. Regardless, this is a currently untouched avenue for prebiotic research and warrants further investigation of fermentation-related effects on cholesterol and obesity-related diseases.

## 7. Conclusions

A great deal of research supports the use of prebiotic compounds for a range of positive health outcomes distant to the gut, particularly for improved bone density, anxiety, hyperammonemia, and blood lipid profile. However, what the current studies lack are a distinct mechanistic link between the metabolism or binding of these compounds and the effects they induce. In some cases, there is a clear, observable connection, such as the ammonia-reducing effects of disaccharides in hepatic encephalopathy patients, but this is relatively rare. More human clinical trials are needed, particularly longitudinal, that have the power to observe subtle changes over the duration of ingestion, as well as carefully controlled animal studies to explain how these effects occur. Given the success of prebiotics in the attenuation of many diseases and improvement of health at distant sites, these food-grade saccharides are becoming key components of a health-promoting diet.

## Figures and Tables

**Table 1:** Summary of findings from human prebiotic interventions on bone health.

Type of Trial	Prebiotic Used	Main Finding	Reference
Randomized, double-blind, placebo-controlled crossover	Inulin/oligofructose mix (Synergy1)	Improved calcium and magnesium absorption and bone turnover in postmenopausal women.	Holloway et al., 2007 [23]
Randomized, double-blind, placebo-controlled crossover	scFOS	scFOS do not improve calcium absorption in postmenopausal women.	Tahiri et al., 2003 [24]
Randomized, double-blind, placebo-controlled	Chicory fructan	Calcium absorption improved by chicory fructan administration in postmenopausal women.	Kim et al., 2004 [25]
Randomized, double-blind, placebo-controlled crossover	TOS	Calcium absorption improved by TOS administration in postmenopausal women.	van den Heuvel et al., 2000 [26]
Randomized, double-blind, placebo-controlled crossover	Lactulose	Calcium absorption improved by lactulose administration in postmenopausal women.	van den Heuvel et al., 1999 [27]
Randomized, double-blind, placebo-controlled crossover	Inulin, FOS, and GOS	Inulin, FOS, and GOS do not affect calcium or iron absorption in healthy adult men.	van den Heuvel et al., 1998 [28]
Randomized, double-blind, placebo-controlled	Inulin-type fructan	Calcium absorption and bone content/density improved by inulin-type fructan administration in teenagers.	Abrams et al., 2005 [29]
Randomized, double-blind, placebo-controlled crossover	Oligofructose and inulin/oligofructose mixture	Calcium absorption improved by inulin/oligofructose, but not oligofructose, administration in adolescent girls.	Griffin et al., 2002 [30]
Randomized, double-blind, placebo-controlled crossover	Oligofructose	Calcium absorption improved by oligofructose administration in adolescent boys.	van den Heuvel et al., 1999 [31]
Randomized, double-blind, placebo-controlled	scFOS	Bone turnover was minimized by scFOS administration in postmenopausal women. No effect on bone mineral density.	Slevin et al., 2014 [32]

**Table 2:** Summary of findings from human prebiotic interventions on memory, attention, learning, and mood.

Type of Trial	Prebiotic Used	Main Finding	Reference
Placebo-controlled crossover	Oligofructose/inulin mixture	Oligofructose/inulin did not affect fatigue, mood, reaction time, attention, or memory after 43 days administration.	Smith, 2005 [59]
Randomized, double-blind, placebo-controlled crossover	Oligofructose/inulin mixture	Oligofructose/inulin improved mood, recognition memory, and recall after 4 h.	Smith et al., 2015 [60]
Randomized, double-blind, placebo-controlled	Non-starch polysaccharides (NSPs, Ambrotose complex)	NSPs improved recall (RAVLT test) and recognition memory and well-being in middle-aged adults.	Best et al., 2009 [61]
Randomized, double-blind, placebo-controlled, between subjects	NSPs (Ambrotose complex)	NSPs improved memory (RAVLT test) acutely in middle-aged adults.	Best et al., 2015 [62]
Cross-sectional, placebo-controlled	Dietary saccharides	Saccharide intake improved self-reported memory in middle-aged adults.	Best et al., 2009 [63]
Randomized, double-blind, placebo-controlled	scGOS/lcFOS/pAOS mixture	Prebiotic mixture did not improve neurodevelopment in preterm infants.	van den Berg et al., 2016 [65]
Randomized, double-blind, placebo-controlled	FOS and B-GOS	B-GOS, but not FOS, reduced salivary cortisol and improved attention in adults.	Schmidt et al., 2015 [71]

**Table 3:** Summary of findings from human prebiotic interventions on immune function.

Type of Trial	Prebiotic Used	Main Finding	Reference
Randomized, double-blind, placebo-controlled, crossover	β2-1 fructans	β2-1 fructans increased blood IL-4, CD282+/TLR2+ myeloid dendritic cells, and a TLR2-mediated immune response in healthy adults.	Clarke et al., 2016 [89]
Randomized, double-blind, placebo-controlled	β2-1 fructans	β2-1 fructans did not affect numbers of blood immune cells or Ig, salivary IgA, or immune activity in healthy adults.	Lomax et al., 2012 [90]
Randomized, double-blind, placebo-controlled	Oligofructose/inulin mixture	Oligofructose/inulin improved antibody response to measles vaccination	Firmansyah et al., 2001 [91]
Randomized, double-blind, placebo-controlled	Oligofructose/inulin mixture	Oligofructose/inulin increased circulating influenza-specific antibodies after vaccination in healthy adults.	Lomax et al., 2015 [92]
Prospective, randomized, double-blind, placebo-controlled	Nutritional formula containing FOS	Nutritional formula with FOS improved influenza vaccine response and reduced symptomatic days in infants.	Langkamp-Henken et al., 2004 [93]
Randomized, double-blind, placebo-controlled	Oligofructose-supplemented cereal	Prebiotic cereal reduced diarrhea-associated fever and medical attention in infants.	Saavedra et al., 1999 [97]
Randomized, double-blind, placebo-controlled	Oligofructose-supplemented cereal	Prebiotic cereal reduced sick days, antibiotic use and febrile seizures in infants.	Tschernia et al., 1999 [98]
Randomized, double-blind, placebo-controlled, prospective	scGOS/lcFOS/pAOS	scGOS/lcFOS/pAOS reduced the development rate of atopic dermatitis in low-risk infants.	Grüber et al., 2010 [100]
Randomized, double-blind, placebo-controlled, prospective	GOS/FOS	GOS/FOS reduced the development rate of atopic dermatitis in high-risk infants.	Moro et al., 2006 [101]
Randomized, double-blind, placebo-controlled, parallel-group	Partially hydrolyzed FOS	FOS reduced antibodies against cow’s milk and increased circulating Treg and plasmacytoid dendritic cells, but did not prevent the development of eczema in infants.	Boyle et al., 2016 [102]
Cross-sectional	FUT2-dependent breast milk oligo-saccharides	FUT2-dependent breast milk oligosaccharides are associated with reduced risk for allergic disease in high-risk infants.	Sprenger et al., 2016 [104]
Randomized, double-blind, placebo-controlled	Probiotic supplement with GOS	Probiotic/prebiotic mixture reduced the occurrence of eczema in high-risk infants.	Kukkonen et al., 2007 [105]
Pretest-posttest	FOS	FOS decreased inflammatory IL-6 expression and phagocytosis activity by granulocytes and monocytes in elderly people.	Guigoz et al., 2002 [112]
Randomized, double-blind, placebo-controlled, crossover	B-GOS	B-GOS increased phagocytosis and NK cell activity while promoting an anti-inflammatory cytokine profile in elderly people.	Vulevic et al., 2008 [113]
Randomized, double-blind, placebo-controlled, crossover	B-GOS	B-GOS increased NK cell activity and circulating IL-10, IL-8, and C-reactive protein, while reducing IL-1β, in elderly people.	Vulevic et al., 2015 [114]

**Table 4:** Summary of findings from human prebiotic interventions on serum lipid profile.

Type of Trial	Prebiotic Used	Main Finding	Reference
Randomized, double-blind, placebo-controlled crossover	Inulin	Inulin reduced blood triacylglycerol and lipogenesis, but did not affect cholesterol in healthy people.	Letexier et al., 2003 [124]
Randomized, double-blind, placebo-controlled	Inulin/oligofructose mixture	Inulin/oligofructose had no effect on plasma lipid profile over 6 months in healthy people.	Forcheron and Beylot, 2007 [125]
Randomized, double-blind crossover	Inulin	Inulin improved serum lipid profile (HDL-cholesterol, triglycerides, and Lipoprotein(a)) in healthy men.	Russo et al., 2008 [126]
Partially randomized crossover	Lactulose and l-rhamnose	Lactulose and l-rhamnose both reduced triacylglycerol levels and synthesis in healthy men. Cholesterol was unaffected.	Vogt et al., 2006 [129]
Randomized, double-blind, placebo-controlled crossover	B-GOS	B-GOS reduced serum cholesterol, triacylglycerol and total:HDL cholesterol ratio in overweight people.	Vulevic et al., 2013 [131]
Randomized, double-blind, placebo-controlled, crossover	B-GOS	B-GOS did not affect serum total or HDL cholesterol in elderly people.	Vulevic et al., 2008 [113]
